# Prevalence of inter-hemispheric asymetry in children and adolescents with interdisciplinary diagnosis of non-verbal learning disorder

**DOI:** 10.1590/S1679-45082016AO3722

**Published:** 2016

**Authors:** Alessandra Bernardes Caturani Wajnsztejn, Bianca Bianco, Caio Parente Barbosa

**Affiliations:** 1 Faculdade de Medicina do ABC, Santo André, SP, Brazil.

**Keywords:** Learning disorders/diagnosis, Child, Adolescent

## Abstract

**Objective:**

To describe clinical and epidemiological features of children and adolescents with interdisciplinary diagnosis of non-verbal learning disorder and to investigate the prevalence of inter-hemispheric asymmetry in this population group.

**Methods:**

Cross-sectional study including children and adolescents referred for interdisciplinary assessment with learning difficulty complaints, who were given an interdisciplinary diagnosis of non-verbal learning disorder. The following variables were included in the analysis: sex-related prevalence, educational system, initial presumptive diagnoses and respective prevalence, overall non-verbal learning disorder prevalence, prevalence according to school year, age range at the time of assessment, major family complaints, presence of inter-hemispheric asymmetry, arithmetic *deficits*, visuoconstruction impairments and major signs and symptoms of non-verbal learning disorder.

**Results:**

Out of 810 medical records analyzed, 14 were from individuals who met the diagnostic criteria for non-verbal learning disorder, including the presence of inter-hemispheric asymmetry. Of these 14 patients, 8 were male.

**Conclusion:**

The high prevalence of inter-hemispheric asymmetry suggests this parameter can be used to predict or support the diagnosis of non-verbal learning disorder.

## RESUMO

**Objetivo:**

Descrever as características clínicas e epidemiológicas de crianças e adolescentes com transtorno de aprendizagem não verbal, e investigar a prevalência de assimetria inter-hemisférica neste grupo populacional.

**Métodos:**

Estudo transversal que incluiu crianças e adolescentes encaminhados para uma avaliação interdisciplinar, com queixas de dificuldades de aprendizagem e que receberam diagnóstico interdisciplinar de transtorno de aprendizagem não verbal. As variáveis avaliadas foram prevalência por sexo, sistema de ensino, hipóteses diagnósticas iniciais e respectivas prevalências, prevalência de condições em relação à amostra total, prevalência geral do transtorno de aprendizagem não verbal, prevalência de acordo com ano escolar, faixa etária no momento da avaliação, principais queixas familiares, presença assimetria inter-hemisférica, dificuldade em aritmética, alterações em visuoconstrução, e principais sinais e sintomas do transtorno de aprendizagem não verbal.

**Resultados:**

Dos 810 prontuários médicos analisados, 14 eram de indivíduos que preencheram os critérios diagnósticos para transtorno de aprendizagem não verbal, incluindo a assimetria inter-hemisférica. Destes 14 pacientes, 8 eram do sexo masculino.

**Conclusão:**

A alta prevalência de assimetria inter-hemisférica sugere que este parâmetro possa ser usado como preditor ou reforçador para diagnóstico de transtorno de aprendizagem não verbal.

## INTRODUCTION

The study of learning difficulties dates back from the 19^th^ century and was motivated by the recognition of reading difficulties unrelated to intellectual ability^(1)^ and sensory *deficits*, level of instruction or learning motivation. The umbrella term “learning disability”^(2,3)^ comprises a group of specific and non-specific disorders affecting reading, writing, math and other basic learning skills. Such disorders are characterized by distinct signs and symptoms manifested by children with learning difficulties, which keep them from achieving their full potential.^(4)^

The generic term “learning disabilities”, proposed by the National Joint Committee on Learning Disabilities,^(5)^ in 1988, refers to a heterogeneous group of disorders characterized by significant difficulties in the acquisition and application of speaking, reading, writing, reasoning and math skills. These lifelong disorders, which are intrinsic to the affected individual, are presumably due to central nervous system dysfunctions. Affected individuals may have concurrent behavior regulation (otherwise known as self-regulation), perception and social competence problems. Another form of learning disability,^(6,7)^ referred to as non-verbal disability, has been proposed to describe children who do not have severe language problems and are able to acquire reading and writing skills, but present with “persistent *deficits* in right-left orientation and difficulties with constructional tasks and arithmetic, whose *deficits* are non-verbal and who are unable to grasp the significance of many aspects of the environment”.

There are no population-based studies addressing the prevalence of non-verbal learning disorder (NVLD). Still, learning disabilities are thought to affect an estimated 10% of school-age children, with 1% prevalence of NVLD in this group,^(8)^ and to account for an estimated 10% of learning-disability-related medical appointments. This study revealed a significant NVLD prevalence. Data presented are expected to inform healthcare and education professionals about the disorder, to prevent false-negative diagnoses.

The major feature of NVLD is the discrepancy between verbal and non-verbal (or performance) intelligence quotients (IQ), controlled by the left and right brain hemispheres, respectively.^(9,10)^ “Attention *deficits* and other more general forms of neurological impairments may actually by masked by information processing *deficits* that occur with advancing age”.^(11)^

Different sets of criteria can be employed to evaluate individuals with NVLD,^(12,13)^ such as the Wechsler Intelligence Scale for Children with verbal IQ greater than 79, and tests aimed to identify *deficits* in tactile and visual perception, complex psychomotor activity and the ability to deal with new materials, which gave rise to the profile matching algorithm developed by the authors.

Non-verbal learning disorder signs and symptoms do not match those of any well-described condition; therefore, difficulties in characterizing this condition remain to this day.^(14)^ Non-verbal learning disorder is a specific condition characterized by difficulties in motor coordination, somatosensory perception, visuospatial cognition, inductive and arithmetic reasoning, cognition and social skills.^(12,15)^

The three major areas of dysfunction in NVLD include motor and visuospatial abilities, organizational skills and social skills.^(16)^

Children and adolescents with NVLD often have good reading and writing skills, but have difficulties with inferential reasoning, reading comprehension and math.^(17)^

Affected children have difficulties understanding cause-effect relationships, poor reasoning and problem solving skills and difficulties understanding complex or abstract ideas, and therefore tend to have executive functioning issues.^(17,18)^

The diagnosis of NVLD is based on the identification of *deficits* in social perception, social judgment and social interaction skills,^(19)^ given such difficulties are secondary to impaired visuospatial development^(20,21)^ and pervade diagnostic strategies based on recognition of significant perception problems, faulty understanding of facial expressions, tone of voice and speaker’s intention.^(22-25)^

Low scores in specific motor performance tests involving both hands suggest bilateral brain involvement and confirm the presence of motor coordination impairments, with worse performance in the right hemisphere compared to the left. Children with NVLD are described as clumsy and uncoordinated.^(25,26)^

Visuospatial *deficits* are the major characteristic of children with NVLD, even in the absence of severe motor problems. Differences between verbal and performance (non-verbal) IQ scores are not a requisite for the diagnosis of NLVD; still this finding has been particularly emphasized in affected children.^(26)^

The neuropsychological model of NVLD^(27)^ accounts for strengths and weaknesses in the child’s skills profile, and describes some *deficits* or skills as essential, while others are thought to be of secondary importance.

Hence, difficulties in the identification and characterization of individuals affected with NVLD remain, as do difficulties in confirmation and quantification of motor, socialization and learning *deficits*, and interhemispheric asymmetry. According to a literature review,^(28)^ interhemispheric asymmetry, *deficits* in fine motor and visuoconstructive coordination, difficulties with visuospatial memory tasks and math, and social emotional impairments are the most significant factors in NVLD. A magnetic resonance imaging study^(29)^ comparing different portions of the corpus callosum revealed significantly smaller splenium in children affected with NVLD compared to children in all other groups studied (control children, children affected with attention *deficit* hyperactivity disorder predominantly inattentive and others, and children with high-functioning autism). Findings in the NVLD group in that study were associated with low performance IQ, but not with low verbal IQ scores.

## OBJECTIVE

To describe clinical and epidemiological features of children and adolescents with interdisciplinary diagnosis of non-verbal learning disorder and to investigate the prevalence of inter-hemispheric asymmetry in this population group.

## METHODS

MEDLINE and ScienceDirect databases were searched using PubMed and Scopus search engines respectively. The keywords “child”, “adolescent” and “non-verbal learning disorder” were used to locate related articles and respective references in PubMed (US National Library of Medicine/National Institutes of Health). Only publications with title or abstract in Portuguese or English were included in the analysis.

This is a cross-sectional study based on data extracted from medical records of patients seen at the *Núcleo Especializado em Aprendizagem da Faculdade de Medicina do ABC* from 2008 to 2014. All patients were children or adolescents submitted to interdisciplinary assessment with learning difficulty complaints and diagnosed with NVLD. The following variables were included in the analysis: sex-related prevalence, educational system (*i.e*., public or private school attendance), initial presumptive diagnosis and respective prevalence, overall NVLD prevalence, NVLD prevalence according to school year, age range at the time of assessment, major family complaints, presence of interhemispheric asymmetry, arithmetic *deficits* and visuoconstruction impairments, and major NVLD signs and symptoms.

The diagnosis was made by a multidisciplinary team via an interdisciplinary assessment protocol applied to students attending private and public elementary schools in the ABC region, State of São Paulo, Brazil. Patients were individually evaluated; cases, medical opinion on the test protocol and interdisciplinary NVLD diagnostic decision making were then discussed in meetings held at the end of the data collection period. This study was approved by the Research Ethics Committee, protocol 471.336, CAAE: 17200213.3.0000.0082.

The interdisciplinary evaluation comprised 14 appointments, as follows: medical history, screening at the Pediatric Neurology unit, three neuropsychology sessions, two speech therapy sessions, two psychology/psychopedagogy sessions, one interdisciplinary clinical discussion session, one report preparation session and a final feedback session with the patient’s legal guardian. The interdisciplinary protocol was systematically applied to all of 810 subjects in the sample. The following instruments were used:^(30-33)^ Attention *deficit* hyperactivity disorder, Children’s Apperception Test, Phonological awareness: instrument of sequential assessment (*Consciência fonológica: instrumento de avaliação sequencial*), Verbal Fluency Test (FAS), House-Tree-Person test, reading comprehension, Rey Complex Figure Test and Recognition Trial, MTA-SNAP-IV scale for evaluation of symptoms of attention-*deficit*/ hyperactivity disorder and oppositional-defiant disorder, Perceived Stress Scale, Thematic Apperception Test, Academic Achievement Test, TIPITI Language Assessment and the third edition of the Weschler Intelligence Scale for Children III.

Non-verbal learning disorder diagnosis was based on the following criteria: bilateral *deficits* in tactile perception, usually more pronounced on the left side of the body; bilateral *deficits* in psychomotor coordination; compromised visuospatial organizational skills; significant difficulties in dealing with new or complex situations and pieces of information; significant *deficits* in nonverbal problem solving, concept building and hypothesis testing; perceptual and temporal orientation distortion; well-developed verbal memorization skills; routine and repetitive verbosity, language content distortion and pragmatic communication impairments; significant *deficits* in arithmetic and reading comprehension with intact isolated word reading ability; significant perceptual, judgment and social interaction *deficits*, often leading to social isolation.

Inclusion criteria were as follows: children and adolescents diagnosed with NVLD regardless of drug treatment, referred for interdisciplinary assessment with learning difficulty complaints. Exclusion criteria comprised central nervous system malformations, neurological syndromes and intellectual disabilities.

Descriptive statistical analysis was based on absolute and relative frequencies and measures of central tendency and dispersion. Statistical analyses were performed using software Epi Info.

## RESULTS

Data on sex-related prevalence, educational system, suspicion of NVLD, NVLD prevalence following interdisciplinary assessment and school grade of the 14 patients diagnosed with NVLD in this sample are given in table 1; initial presumptive diagnosis and respective prevalence are given in table 2.

**Table 1:** Sex-related prevalence, educational system, school year, referral initiative and initial non-verbal learning disorder suspicion

	Higher incidence	Lower incidence
	n (%)	n (%)
Sex-related prevalence	589 boys (72.71)	221 girls (27.28)
Educational system	679 private (83.82)	131 public (16.17)
Initial NVLD suspicion	808 not suspected (99.76)	2 suspected (0.24)
NVLD prevalence	Non-NVLD 806 (98.28)	14 cases (1.72)
School stage	12 cases in	2 cases in
	High School (85.71)	Primary School (14.29)
Gender prevalence in the NLVD sample	8 boys (57.15)	6 girls (42.85)
Referral for interdisciplinary assessment	11 cases referred by health professionals (78.57)	3 cases referred by other professionals (21.43)

NVLD: non-verbal learning disorder.

**Table 2:** Initial presumptive diagnoses made by referring professionals, with complaints of learning disabilities and respective prevalence

Autism spectrum disorder	1
Intellectual disability	1
Non-verbal learning disorder	2
Attention *deficit/ hyperactivity* disorder predominantly inattentive	4
Learning disability	6
Number of cases diagnosed with non-verbal learning disorder	14

Family complaints associated with the initial presumptive diagnosis were major factors in comorbidity identification, as shown in table 3.

**Table 3:** Family complaints associated reported

Major family complaints	Incidence
Autism spectrum disorder	1
Immaturity	1
Anxiety	1
Inattention	5
Motor	9
Socialization	11
Ingenuity	13
Shyness	14
Communication	14
Learning	14

Interhemispheric asymmetry was a common finding in NVLD patients (Table 4).

**Table 4:** Interhemispheric asymmetry

Non-verbal learning disorder	14
Intelligence quotient >69	14
Borderline total intelligence quotient ≤79	2
Borderline verbal intelligence quotient >79	14
Performance intelligence quotient dysfunction	2
Borderline performance intelligence quotient	7
Performance intelligence quotient below average	3
Average performance intelligence quotient	2
Interhemispheric asymmetry	14

As an aside, arithmetic and Rey Complex Figure Test and Recognition Trial test results are shown in table 5.

**Table 5:** Arithmetic and Rey Complex Figure tests

Arithmetic	Incidence	Rey figure	Incidence
Average	4	Average	1
Below average	5	Below average	8
Borderline	5	Borderline	3
Dysfunction	0	Dysfunction	2

## DISCUSSION

Non-verbal learning disorder research has seen significant advances since 1964.^(6)^ Following a similar path, this study addressed the definition of NVLD-specific criteria for clinical characterization of NVLD.

Non-verbal learning disorder has been described^(5)^ as a learning disability subtype and is thought to be a form of pervasive developmental disorder.^(34)^ These possibilities have not been listed in the fifth edition of Diagnostic and Statistical Manual of Mental Disorders (DSM-5), published in 2013,^(35)^ and remain to be confirmed.

Visuospatial impairments, tactile and motor *deficits* are the major criteria for learning disorder diagnosis.^(6,20,21)^

One of the most important NVLD diagnostic criteria is the presence of interhemispheric asymmetry in association with low performance intelligence quotient scores and attention *deficits*, which tend to decrease with neurological development.^(9,10,29)^

Other criteria, such as right-left orientation issues, construction and nonverbal arithmetic difficulties, and difficulties to grasp the meaning of many aspects of the environment have also been described.^(5)^

The following criteria were proposed by Hale,^(14)^ *deficits* in motor coordination, perceptual and somatosensory *deficits*, visuospatial impairments, difficulties with inductive and arithmetic reasoning, and impaired cognitive and social skills.

Secondary and tertiary *deficits* associated with NVLD were described and would include delayed acquisition or failure to acquire the necessary ability to perform tasks, such as riding a bicycle or tying shoe laces, graphic renditions with infantile characteristics (*e.g*., inconsistent strokes), impaired spatial organization, difficulties memorizing multiplication tables and executing complex arithmetic operations (such as algorithms), shyness, social awkwardness, ingenuity, little or no malice, faulty recognition of implicit aspects in social interactions (*i.e*., sarcasm, irony and metaphors), incoordination and clumsiness.

Data analysis revealed similar gender-related prevalence in the sample studied (8 boys *versus* 6 girls affected with NVLD). Similar data have been reported elsewhere.^(25)^

The relationship between NVLD and educational system has not been described in literature. This study revealed higher NVLD incidence in private schools; however, this finding might be explained by the fact that children in this sample attended predominantly private schools.

The prevalence of NVLD in the overall child/adolescent population has not been reported; still, 1% of patients with learning disorders are thought to suffer from NVLD. A 1.72%-prevalence of NVLD was documented in this study.

As regards the initial evaluation by referring professionals, this study revealed that 64.28% of patients were referred by healthcare professionals, particularly physicians.

In this study, only 14.28% of 14 patients diagnosed with NVLD were suspected of the disorder. Out of 810 cases included in the sample, 12 were false negative, and there were no false positive results.

Data analysis revealed that 85.52% of patients diagnosed NVLD were in high school at the time of interdisciplinary assessment. Similar findings have been reported in literature.^(14)^ Data of this study also suggest that affected patients tend seek advice around the beginning of the middle school cycle; increased interpretation, logical thinking and socialization demands in this phase tend to exacerbate NVLD signs and symptoms.

Age group analysis revealed a trend towards diagnosis at 12 to 13 years of age, again coinciding with the beginning of the high school cycle.

Three significant aspects related to family complaints reported during anamnesis were emphasized in this study. Shyness, learning and communication *deficits* were reported in all 14 NVLD cases. Naive thoughts and socialization difficulties were reported by 13 and 11 families, respectively. Nine families reported fine, global or gross motor *deficits*.

Awkwardness, faulty understanding of implicit aspects and difficulties associated with inferential reasoning, interpretation, production of spontaneous or direct speech, receptivity and prosody of speech were present in all 14 NVLD cases submitted to interdisciplinary assessment in this study.

As in previous studies,^(9)^ interhemispheric asymmetry with low performance intelligence quotient scores and total intelligence quotient scores above 69 was a consistent finding in NVLD patients in this sample.

Difficulties with arithmetic, logical reasoning and visuospatial organization have been reported in earlier NVLD studies.^(9)^ Patients in this sample scored average, below average or borderline (4, 5 and 5 out of 14 cases, respectively) in the arithmetic subtest of Weschler Intelligence Scale for Children III. Rey Complex Figure Test and Recognition Trial scores of 14 patients were as follows: one average, eight below average, three borderline and two dysfunction. Results of both tests were consistent with findings reported by other researchers.^(26)^

As regards the psychosocial impact on NVLD, given their apparently normal linguistic performance, affected children and adolescents are often expected to perform beyond their actual abilities. Such unrealistic expectations inevitably lead to frustration. The psychosocial impacts of NVLD may be exacerbated by comorbidities, such as externalizing (preschool age children) and internalizing disorders (from preadolescence).

## CONCLUSION

This study revealed a significant prevalence of non-verbal learning disorder and highlighted relevant aspects of the disease. Data presented provide significant contribution to the definition of non-verbal learning disorder diagnostic criteria. Confirmation of interhemispheric asymmetry in all 14 patients diagnosed with non-verbal learning disorder in this sample suggests this parameter can be used to predict or support the diagnosis of the condition.

Growing evidences of non-verbal learning disorder suggest the condition deserves closer attention on the part of health and education professionals, as the knowledge acquisition process is an arduous journey for individuals with learning and communication.

Non-verbal learning disorder signs and symptoms overlap with those of other conditions, such as social pragmatic communication and autism spectrum disorders. Therefore, disclosure of reliable data on non-verbal learning disorder will certainly contribute to faulty diagnosis mitigation and provision of better management guidelines, with positive impacts on the quality of life of affected patients and their families.

The study of the trajectory of patients affected with non-verbal learning disorder and the characterization of disease-related impacts comprise a highly relevant topic for future research aimed at preventing the development of comorbidities, such as anxiety and depression.

Investigation of the impact of bullying against individuals with non-verbal learning disorder is vital, given this form of suffering is a major triggering factor for anxiety, depression, phobia, social isolation and poor academic performance.

Further research into the relations between teaching and learning and the development of alternative evaluation methods adapted to individuals affected with non-verbal learning disorder is warranted, given they tend to learn more through repetition than intuitive or inductive experience.

Family disease history investigation may aid in the identification of potential underlying disease mechanisms, as may the investigation of maternal prenatal, perinatal and postnatal care. The recognition of biomarkers, genetic and hereditary factors may also promote the development of case-specific interventions and more accurate prognostication. Research data can be applied to the development of prevention strategies and actions aimed to decrease the socioeconomic impacts of non-verbal learning disorder on the public and private healthcare systems.
